# The biocide triclosan as a potential developmental disruptor in *Mytilus* early larvae

**DOI:** 10.1007/s11356-023-29854-2

**Published:** 2023-09-20

**Authors:** Teresa Balbi, Angelica Miglioli, Michele Montagna, Davide Piazza, Beatrice Risso, Remi Dumollard, Laura Canesi

**Affiliations:** 1 Department of Earth, Environmental and Life Sciences-DISTAV, University of Genoa, Genoa, Italy; 2 National Biodiversity Future Center, 90133 Palermo, Italy; 3 UMR7009 Laboratoire de Biologie du Développement, Sorbonne Université/CNRS, Institut de La Mer, Villefranche-Sur-Mer, France

**Keywords:** Biocides, Mussel, Early larvae, Gene expression, Shell biomineralization, Developmental disruption

## Abstract

The broadly utilized biocide triclosan (TCS) is continuously discharged in water compartments worldwide, where it is detected at concentrations of ng-µg/L. Given its lipophilicity and bioaccumulation, TCS is considered potentially harmful to human and environmental health and also as a potential endocrine disruptor (ED) in different species. In aquatic organisms, TCS can induce a variety of effects: however, little information is available on its possible impact on invertebrate development. Early larval stages of the marine bivalve *Mytilus galloprovincialis* have been shown to be sensitive to environmental concentrations of a number of emerging contaminants, including EDs. In this work, the effects of TCS were first evaluated in the 48 h larval assay in a wide concentration range (0.001–1,000 μg/L). TCS significantly affected normal development of D-veligers (LOEC = 0.1 μg/L; EC_50_ = 236.1 μg/L). At selected concentrations, the mechanism of action of TCS was investigated. TCS modulated transcription of different genes involved in shell mineralization, endocrine signaling, ceramide metabolism, and biotransformation, depending on larval stage (24 and 48 h post-fertilization-hpf) and concentration (1 and 10 μg/L). At 48 hpf and 10 μg/L TCS, calcein staining revealed alterations in CaCO_3_ deposition, and polarized light microscopy showed the absence of shell birefringence due to the mineralized phase. Observations by scanning electron microscopy highlighted a variety of defects in shell formation from concentrations as low as 0.1 μg/L. The results indicate that TCS, at environmental exposure levels, can act as a developmental disruptor in early mussel larvae mainly by interfering with the processes of biomineralization.

**Supplementary Information:**

The online version contains supplementary material available at 10.1007/s11356-023-29854-2.

## Introduction

Triclosan (2,4,4’-trichloro-2’-hydroxydiphenyl ether, TCS) is an antimicrobial agent present in a variety of personal, household, and healthcare products, as well as in plastic leachates (https://www.epa.gov/ingredients-used-pesticide-products/triclosan). Due to its incomplete removal by conventional wastewater treatment, TCS is continuously discharged in aquatic compartments worldwide, where it is present at concentrations of ng-µg/L. Given its strong lipophilicity and bioaccumulation, TCS is potentially harmful to human and environmental health (Chen et al. 2023; Milanović et al. 2023). TCS is also considered as a potential endocrine disruptor (ED) in different species, and it is included in list II: substances under evaluation for ED assessment under REACH or the Biocidal Products Regulation (https://echa.europa.eu/ed-assessment).

Based on short-term ecotoxicity tests, TCS is classified as very toxic to aquatic life (EC_50_ ≤ 1 mg/L) (https://echa.europa.eu/substance-information/-/substanceinfo/100.020.167). TCS has been reported to be accumulated and to induce multiple effects in aquatic organisms (reviewed in Kumar et al. 2021). TCS and its metabolite methyl-TCS have been shown to affect embryonic development of *Danio rerio* and *Paracentrotus lividus* (Macedo et al. 2017).

In bivalve mollusks, TCS has been shown to affect the immune function (Canesi et al. 2007), to induce changes in a number of biomarkers from molecular to organism level (Matozzo et al. 2012; Parolini et al. 2013), and to affect behavior, metabolic and heart rate, and gene and protein expression (Riva et al. 2012; Goodchild et al. 2016). More recently, the impact of TCS together with increased temperature has been investigated in a global change scenario (Freitas et al. 2019; Costa et al. 2020; Maynou et al. 2021). Data are also available on the effects of TCS on bivalve early development, evaluated by the standard embryotoxicity assay (Cortez et al. 2012; Di Poi et al. 2018; Tato et al. 2018; Rolton et al. 2022). However, limited information is available on the mechanisms of action of TCS in embryo-larval development of bivalves.

In the mussel *Mytilus galloprovincialis*, early larval stages have been shown to be sensitive to environmental concentrations of a number of emerging contaminants, including EDs like bisphenol A (BPA) and tetrabromobisphenol A (TBBPA) (Balbi et al. 2016; Miglioli et al. 2021a, 2021b), different types of pharmaceuticals (Balbi et al. 2018; Franzellitti et al. 2019; Canesi et al. 2022) and nano-microplastics (Balbi et al. 2017; Capolupo et al. 2018), with main effects on shell biogenesis and development of the neuroendocrine system.

In this work, the possible mechanisms of action of TCS on early larval development of *M. galloprovincialis* were investigated. The effects of TCS, in a wide concentration range encompassing environmental exposure levels (from 0.001 to 1,000 μg/L), were first evaluated by the 48-h larval assay. At selected concentrations (1 and 10 μg/L), transcriptional profiles of different groups of genes involved in multiple processes (shell biogenesis, antioxidant/xenobiotic responses, neuroendocrine signaling, sphingolipid metabolism, and apoptosis/differentiation) were assessed across early development (from eggs to 24 and 48-h post-fertilization). Shell formation at 48 hpf was also analyzed by calcein fluorescence, polarized light microscopy (PLM), and scanning electron microscopy (SEM).

## Methods

### Mussels, gamete collection, and larval assay

Sexually mature mussels (*M. galloprovincialis* Lam.), obtained from an aquaculture farm in the Ligurian Sea (La Spezia, Italy) between November 2021 and March 2022, were acclimatized in static tanks containing aerated artificial sea water (ASW) (ASTM 2004), pH 7.9–8.1, 36 ppt salinity (1 L/animal), at 16 ± 1 °C. Gamete collection by spontaneous spawning and fertilization was performed as previously described (Fabbri et al. 2014; Balbi et al. 2016, 2017, 2018; Miglioli et al. 2019). Fertilization was carried out with an egg:sperm ratio 1:10 (fertilization success > 85%), and larvae were grown at a density of 200 larvae/mL in 96-well plates for the 48 h larval assay, 24-well plates for morphological analyses, and 6-well plates for qPCR.

The 48-h larval assay (ASTM 2004) adapted to 96 microwell plates (Fabbri et al. 2014; Balbi et al. 2016, 2018) was performed. Briefly, at 30-min post-fertilization, aliquots of 20 μL of a 10 × solution of TCS (obtained from a 1 g/L stock solution in ethanol), suitably diluted in filter sterilized ASW, were added to fertilized eggs to reach the desired nominal final concentrations (from 0.001 to 1,000 μg/L) in a 200-μL final volume. Control samples in ASW and samples in ASW added with vehicle (ethanol, maximal final concentration 0.01% v/v) were run in parallel. Samples from 6 independent parental pairs were utilized. At 48 hpf, samples were fixed with 4% buffered formalin and examined by and inverted Olympus IX53 microscope (Olympus, Milano, Italy) at 40 × , equipped with a CCD UC30 camera and a digital image acquisition software (cellSens Entry) by an operator blind to the experimental conditions. A larva was considered normal when the shell was D-shaped (straight hinge) and the mantle did not protrude out of the shell, and malformed if had not reached the stage typical for 48 hpf (trochophore or earlier stages) or when some developmental defects were observed (concave, malformed or damaged shell, protruding mantle). The recorded endpoint was the percentage of normal D-larvae (D-veligers) in each well respect to the total, including malformed larvae and pre-D stages (acceptability of test results based on controls with normal D-shell larvae > 75%). Even at the highest concentration, ethanol was ineffective (data not shown).

### Shell size analysis

Light microscopy images of D-veligers at 48 hpf in control larvae and larvae exposed to 10 and 100 μg/L TCS were analyzed for shell length (the maximum anterior–posterior dimension of the shell parallel to the hinge line) and height (the maximum dorsal–ventral dimension perpendicular to the hinge) using ImageJ software as previously described (Balbi et al. 2018). Samples from 4 independent parental pairs were utilized.

### RNA extraction and qPCR analysis

Control larvae and larvae exposed to 1 and 10 μg/L TCS grown in 6 well plates were collected at 24 and 48 hpf by a nylon mesh (0.22-μm pore filter). Three wells for each condition were pooled in order to obtain approximately 6000 larvae/replicate. The larval suspension was centrifuged at 800 × g, for 10 min at 4 °C and the pellets lysed in 1-mL TRI Reagent (Sigma Aldrich, Milan, Italy). All the subsequent analyses (RNA extraction, retro-transcription, and qPCR) were performed following Balbi et al. (2016, 2017, 2018). Primers employed for qPCR analysis are reported in Table S1. HEL and EF-α1 were utilized as reference genes (Balbi et al. 2016). A comparative *C*_*T*_ method was utilized for calculations of relative expression of target mRNAs (Schmittgen and Livak 2008). Data are reported as relative expression (mean ± SD) with respect to control samples within each larval stage. Samples from 4 independent parental pairs were utilized.

### Calcein staining

Shell calcification of control larvae and larvae exposed to TCS (10 μg/L) at 48 hpf was evaluated by calcein staining as previously described (Miglioli et al. 2019, 2021a, 2021b). The Ca^2+^-dependent fluorophore calcein (Sigma Aldrich, Lyon, France) was added to fertilized eggs (final concentration 1 mM in 0.01% dimethyl sulfoxide-DMSO). At 48 hpf, larvae were washed three times in ASW to remove the excess dye, fixed in 4% paraformaldehyde (PFA) in ASW, and immediately imaged with a Leica TCS SP8 Confocal Microscope (Leica, France; Exc: 488 nm/Em: 520–560 nm) to visualize the calcified shell in green.

### Polarized light microscopy (PLM)

Samples from control larvae and larvae exposed to TCS (10 μg/L) at 48 hpf were collected on 0.22-μm filters, washed four times with deionized water to remove excess salts, and dried at 60 °C for 30 min. Observations were carried out on glass slides by a polarized light microscope (OLYMPUS BX-41, 40 ×) as previously described (Balbi et al. 2017). Images were acquired by an Olympus Color view II and digitalized by the Olympus Color view II Bund Cell B.

### Scanning electron microscopy (SEM)

For SEM analysis, control larvae and larvae exposed to 0.1, 1, 10, and 100 μg/L TCS at 48 hpf were processed as previously described (Balbi et al. 2016, 2018), fixed in 3% glutaraldehyde in ASW, pooled from 24 wells, placed onto Whatman 0.22-μm filters, dehydrated in an ascending series of ethanol washes (50–80–90–100%), and air-dried. Then, samples were sputter-coated with gold and observed at 20 kV with a Vega3-Tescan scanning electron microscope.

### Data analysis

Data from the 48-h larval assay, expressed as mean ± SD of 6 experiments carried out in 3 replicate wells, were analyzed by ANOVA plus Dunnet’s post hoc test. The EC_50_, as the concentration causing 50% reduction in the embryogenesis success (at 95% confidence intervals C.I.), was calculated by PRISM 5 software (GraphPad Prism 5 software package, GraphPad Inc.). LOEC (lowest effect concentration) was evaluated by the Mann–Whitney *U* test.

Data on gene transcription, obtained from 4 independent RNA samples, are expressed as mean ± SD. Statistical differences were evaluated with respect to controls (*p* < 0.05, Mann–Whitney *U* test).

## Results

### Effects of TCS on larval development

**Fig. 1:**
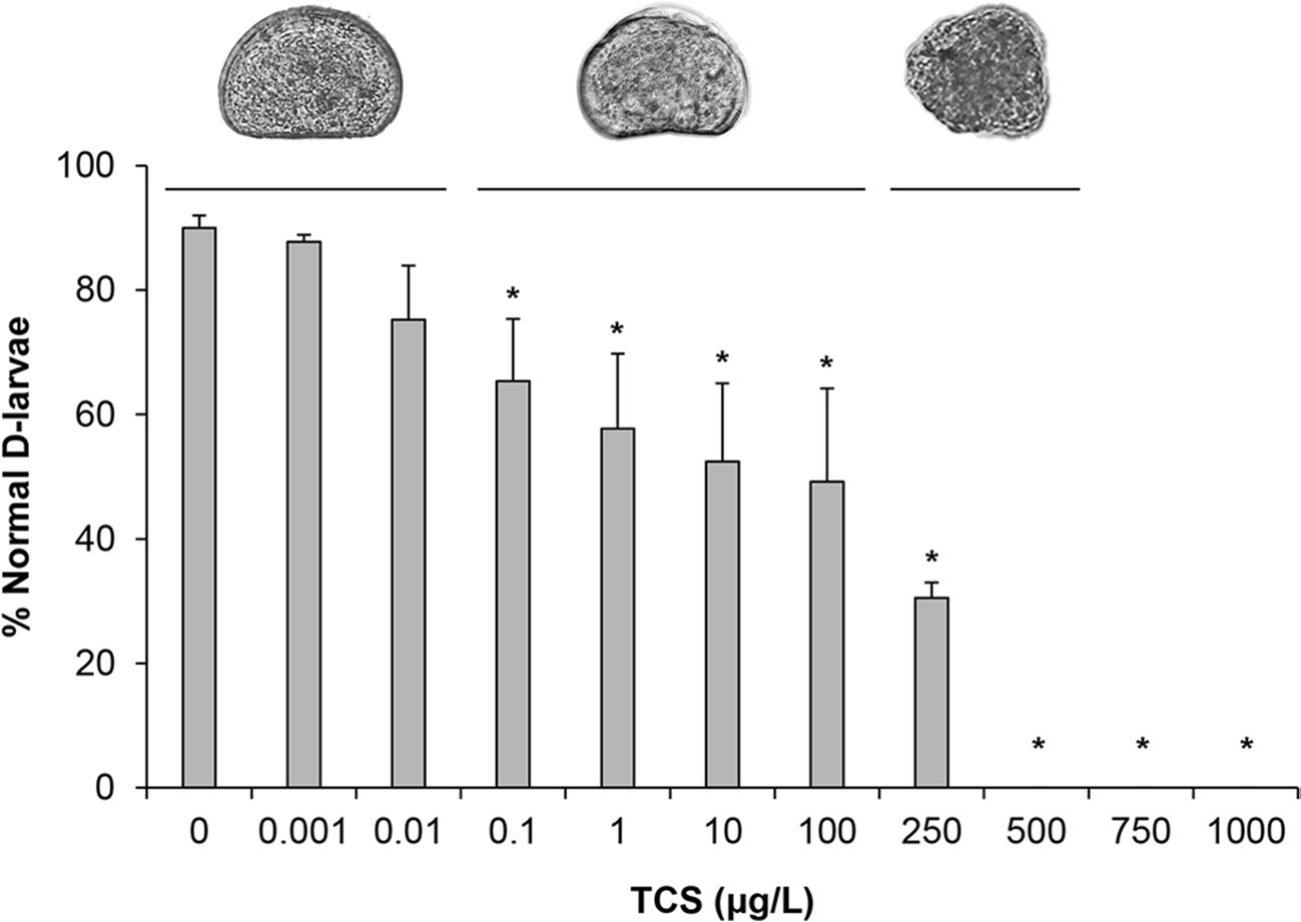
Effects of TCS on *M. galloprovincialis* development in the 48-h larval assay. Data, representing the mean ± SD of 6 experiments in triplicate, were analyzed by ANOVA plus Dunnett’s post hoc test (*p* < 0.01). Representative images of larval phenotypes observed at different concentration ranges are reported

The results of the 48-h assay (Fig. 1) show that TCS significantly affected larval development from 0.1 μg/L (− 26% with respect to controls, *p* < 0.01). Further, small decreases in the percentage of normal D-larvae were progressively observed in a wide concentration range (from − 34 to − 44% at 1 to 100 μg/L, respectively), with malformed larvae representing the dominant phenotype. A sharper decline was observed at 250 μg/L (− 65%); in these conditions, only immature larvae (mainly pre-veligers and some trochophore) were detected, indicating developmental delay. Highest concentrations of TCS (from 750 μg/L) completely prevented larval development. A resulting EC_50_ of 236.1 μg/L (95% C.I. 139–498.2) was obtained.

Shell size was evaluated in D-larvae of control samples and in samples exposed to TCS 10 and 100 μg/L, and the results are shown in Fig. S1. In control samples, mean shell length and width were 93.94 ± 2.85 µm and 67.68 ± 2.64 µm, respectively. Although TCS exposure did not result in significant changes in mean shell size, distinct distribution curves were observed among control and TCS-exposed samples (Fig. S1), indicating a progressive shift toward lower length and width values at increasing TCS concentrations.

### Effects of TCS on gene expression

The level of RNA transcripts for genes involved in different biological processes: shell biogenesis, neuroendocrine signaling, antioxidant/biotransformation processes, sphingolipid (ceramide) metabolism, and apoptosis/proliferation, was evaluated by qPCR (Tab. S1). Data on basal expression in larvae at 24 and 48 hpf with respect to eggs indicate progressive upregulation across development for most genes (Fig. S2).

**Fig. 2:**
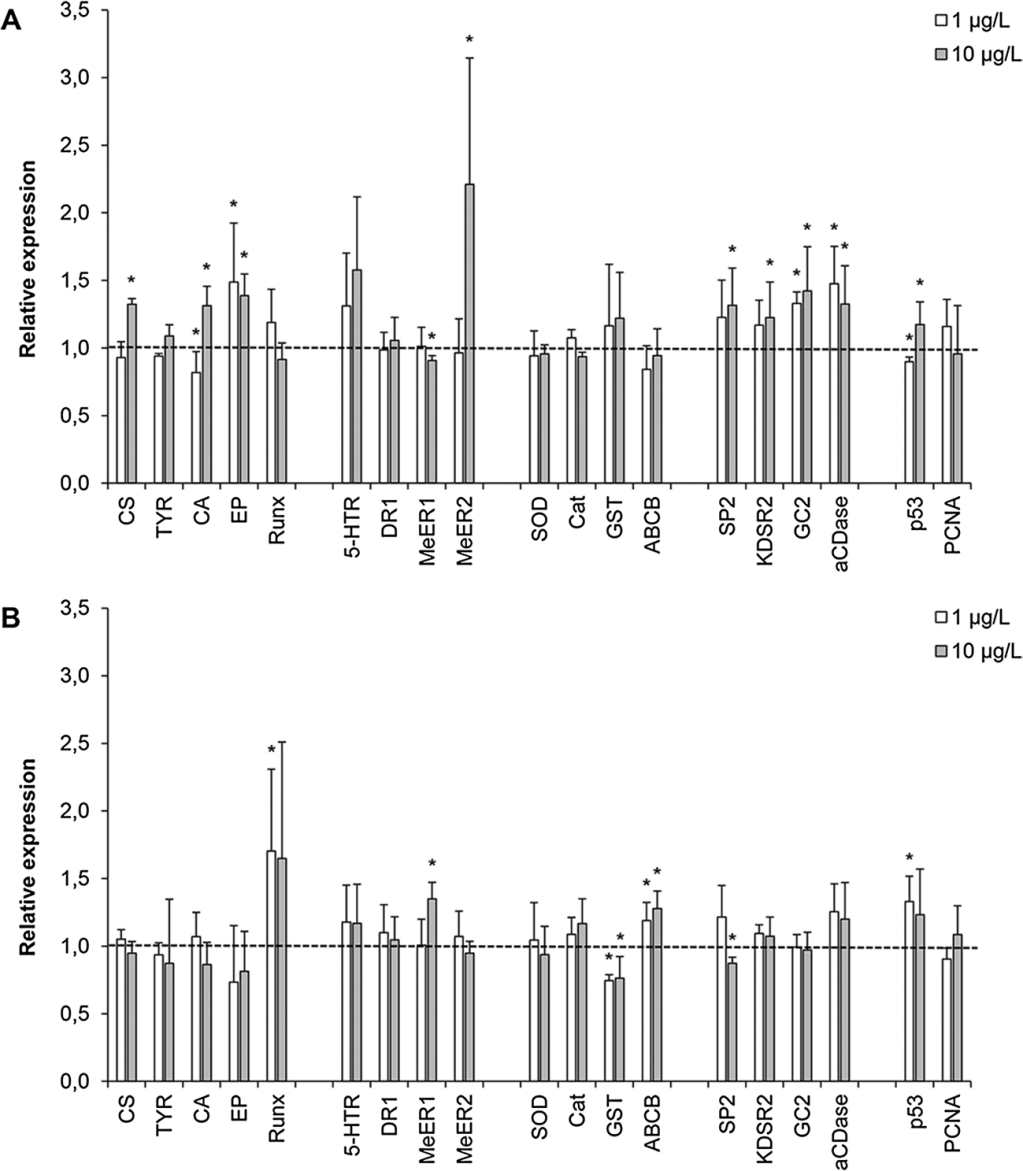
Effects of TCS (1 and 10 μg/L) on gene transcription at 24 (**A**) and 48 (**B**) hpf. Data are reported as the mean ± SD of relative expression with respect to controls (*n* = 4). *p < 0.05, Mann–Whitney *U* test

The effects of TCS (1 and 10 µg/L) on gene transcription at 24 and 48 hpf are reported in Fig. 2 (A and B, respectively).

#### Shell biogenesis

mRNA levels of genes involved in shell organic matrix deposition (chitin synthase-CS; tyrosinase-TYR) and calcification (carbonic anhydrase-CA; extrapallial protein-EP) (Miglioli et al. 2021b), as well as of Runx, a transcription factor involved mammalian bone formation and in larval shell morphogenesis (Kapsenberg et al. 2022 and refs. therein), were evaluated. Expression of TYR was unaffected by TCS exposure, whereas a small increase in CS expression was observed in 24 hpf larvae at 10 µg/L (+ 30% with respect to controls). At 24 hpf, TCS induced upregulation of EP at 1 µg/L and of both EP and CA at 10 µg/L (+ 50, + 40, and + 30%, respectively). At 48 hpf, both concentrations increased transcription of Runx (+ 70%).

#### Neuroendocrine signaling

TCS did not significantly affect expression of the monoamine receptors 5-HTR and DR1. Significant increases in expression of MeER1 and MeER2, representing *Mytilus* estrogen related receptor and estrogen receptor, respectively (Nagasawa et al. 2015), were induced by 10 µg/L TCS at 24 hpf for MeER2 (+ 120%) and at 48 hpf for MeER1 (+ 35%).

#### Antioxidant and biotransformation-related genes

TCS did not affect expression of SOD and CAT in any experimental condition. In contrast, at 48 hpf, both concentrations of TCS induced downregulation of GSH transferase (GST) and upregulation of the xenobiotic transporter ABCB (about − 25% and + 25%, respectively, compared to controls).

#### Sphingolipid metabolism

Expression of genes involved in ceramide biosynthesis (serine palmitoyltransferase-1, 3-ketodihydrosphingosine reductase), metabolism (ceramide glucosyltransferase), and breakdown (acid ceramidase) were evaluated (Timmins-Schiffman and Roberts 2012; Balbi et al. 2022). At 24 hpf, aCDase and GC2 were significantly upregulated by both TCS concentrations (+ 48 and + 32% for aCDase, and + 30 and 40%, for GC2 at 1 and 10 µg/L, respectively). At this stage, small increases in expression of SP2 and KSDR2 were also induced by 10 µg/L TCS (+ 30 and + 20%, respectively). At 48 hpf, only SP2 was slightly downregulated (− 20%).

#### Apoptosis/proliferation

Expression of p53 and PCNA was unaffected by TCS exposure.

### Shell calcification

**Fig. 3:**
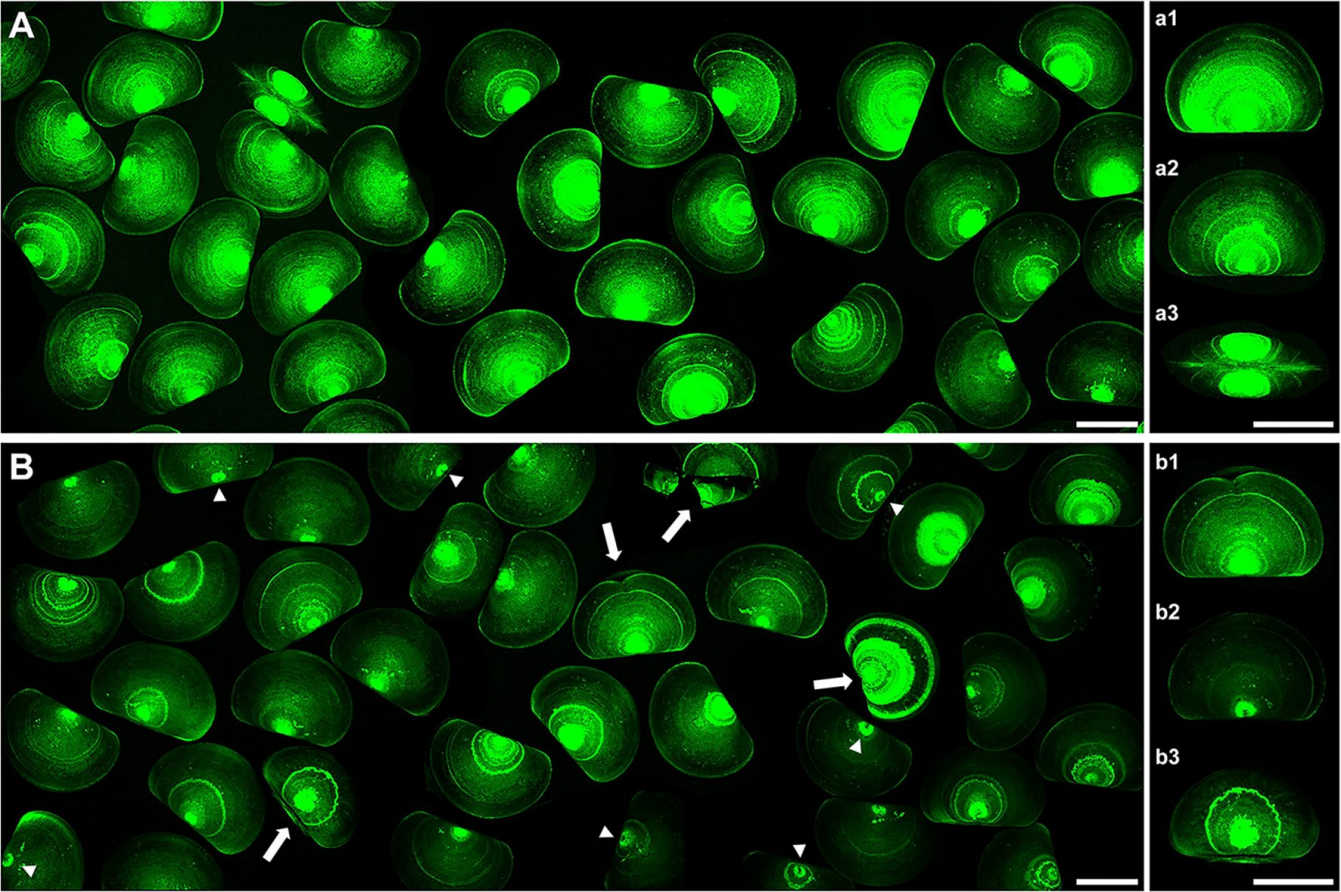
Representative images of calcein staining of control and TCS-exposed larvae (10 μg/L) at 48 hpf evaluated by confocal microscopy. Left panels (**A** and **B**): general view. Right panels: details of representative individuals in each experimental group (a1–a3; b1–b3). **A** Control larvae, showing normal D-veligers characterized by an extensive fluorescent signal (green). a1 and a2: calcification covering a large part of the larval body and concentric accretion rings (lateral view); a3: straight and closed hinge (dorsal view). **B** TCS-exposed larvae, showing a generally lower fluorescence signal, malformed and broken shells (arrows) and absence of calcification in the center of the shell (arrowheads). b1: malformed shell; b2: lower calcification and absence of signal in the center of the growing valvae (lateral views); b3: absence of calcification along the hinge (latero-dorsal view). Scale bars: 50 μm

Fluorescent calcein staining was utilized to visualize CaCO_3_ deposition in 48 hpf larvae as previously described (Kapsenberg et al. 2018; Miglioli et al. 2019, 2021a, 2021b), and representative confocal images are reported in Fig. 3. In control larvae (Fig. 3 A), extensive shell calcification was observed (green), with concentric accretion rings, including the hinge region (see details in insets a1, a2, and a3). In samples exposed to TCS (10 μg/L) (Fig. 3 B), the calcein signal was generally lower, and absent in the hinge region (insets b1, b2, and b3), indicating decreased calcification. Moreover, many shells were characterized by the absence of CaCO_3_ deposition in the center of the growing valvae (inset b2).

### Shell mineralization

**Fig. 4:**
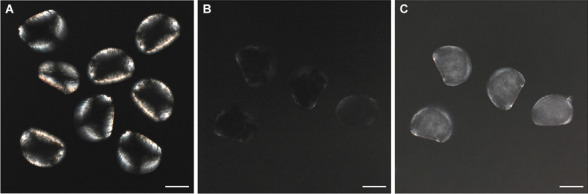
Effects of TCS (10 μg/L) on shell mineralization of *Mytilus* larvae at 48 hpf evaluated by polarized light microscopy. **A** Control larvae showing evident shell birefringence consistent with a fraction of crystalline shell material; **B** larvae exposed to TCS (10 μg/L) where no birefringence could be observed. **C** TCS-exposed larvae, light microscopy. Scale bars: 50 μm

The effects of TCS (10 µg/L) on shell mineralization at 48 hpf were investigated by PLM as previously described (Balbi et al. 2017), evaluating birefringence due to the mineral phase (Weiss et al. 2002). Representative images (Fig. 4) show that control D-veligers exhibited an evident birefringence over the shell area, indicating CaCO_3_ mineralization (Fig. 4 A). In contrast, no birefringence was observed in shells of larvae exposed to 10 μg/L TCS (Fig. 4 B), indicating the absence of CaCO_3_ in the mineralized form.

### Scanning electron microscopy (SEM)

**Fig. 5:**
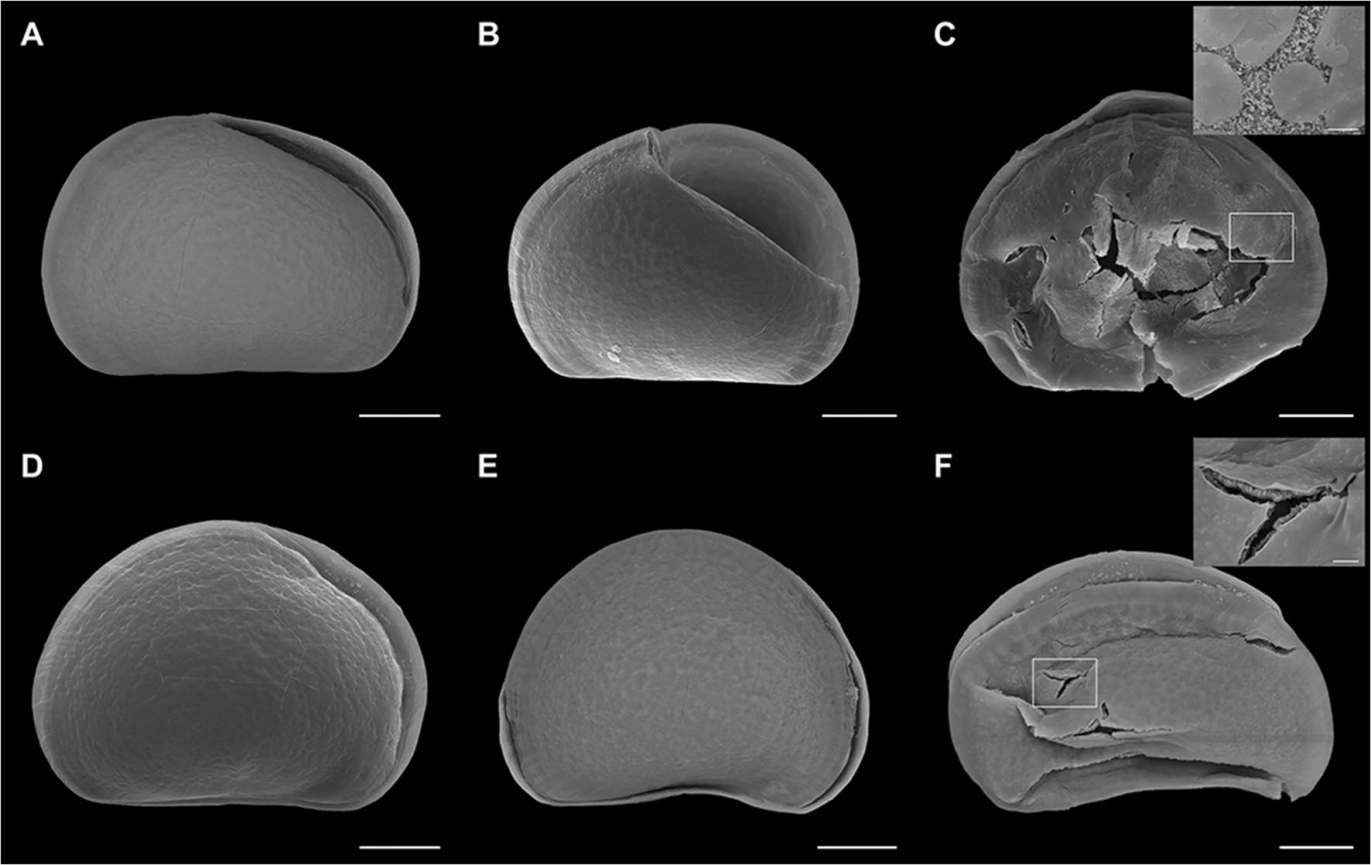
Effects of TCS (10 and 100 μg/L) on shell morphology of *Mytilus* larvae at 48 hpf, evaluated by SEM. **A**, **B** Control D-veligers, with straight hinge, symmetric valvae, and uniform, nearly smooth surface. **C**, **D** larvae exposed to 10 μg/L TCS, with straight hinge but showing cracks on shell surface, asymmetric valvae, and irregular surfaces. **E**, **F** Larvae exposed to 100 μg/L TCS, with (**E**) straight hinge, but asymmetric valvae and irregular surface and (**F**) malformed, smaller shells, with thicker hinge and margins. Scale bars: 20 μm

**Fig. 6:**
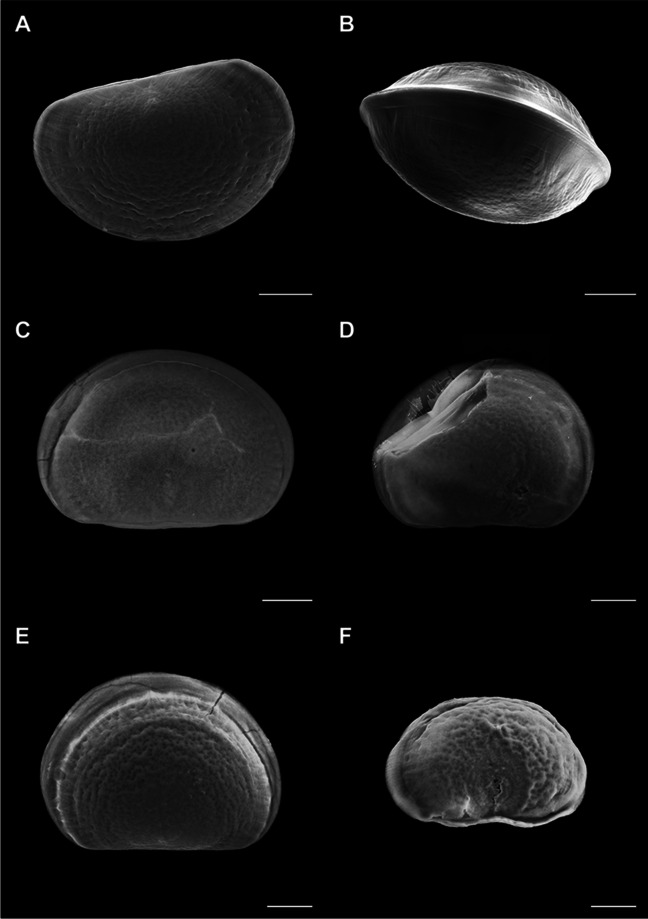
Effects of TCS (0.1 and 1 μg/L) on shell morphology of *Mytilus* larvae at 48 hpf, evaluated by SEM. **A–C** TCS 0.1 μg/L: **A**, **B** asymmetric and incomplete valvae, but straight hinges (**C**) thin, fractured shell, revealing the presence of irregular mineralization patches in the surface, containing nanosized granules (inset). **D–F** TCS 1 μg/L: **D** asymmetric valvae, **E** malformed shell with convex hinge, **F** malformed larvae, with fractures revealing the thinness of the mineralized shell (inset). Scale bars: 20 μm; in insets, scale bars: 2 μm

Shells of control samples and samples exposed to different concentrations of TCS at 48 hpf were observed by SEM, and the results are reported in Figs. 5 and 6. Figure 5 A and B shows representative images of normal D-veligers, whose shells were characterized by a straight hinge, symmetric valvae, and rather smooth surface as previously described (Balbi et al. 2016, 2017, 2018). In samples exposed to TCS (10 and 100 μg/L), D-shells, despite showing rather straight hinges (a morphological parameter associated with the normal D-larval phenotype), had asymmetric or incomplete valvae, as well as irregular and cracked surfaces (Fig. 5 C and E). Other shells were broken or malformed, with irregular and scarred surfaces (Fig. 5 D and F). At 100 µg/L, D-veligers of smaller size were also observed, characterized by a rough surface and thickening of both hinge and margins (Fig. 5 F).

Also at lower TCS concentrations (0.1 and 1 μg/L), D-shells with normal hinges but with asymmetric valvae were detected (Fig. 6 A and D), as well as incomplete and malformed shells (Fig. 6 B and E, respectively). Moreover, extremely thin and broken shells were observed, from concentrations as low as 0.1 µg/L (see Fig. 6 C and F, inset). These samples also revealed incomplete mineralization, as indicated by the presence of irregular patches on the surface and of granules of 100–200-nm diameter (Fig. 5 D, inset), resembling amorphous calcium carbonate (ACC).

## Discussion

The results indicate that TCS can act as a potential developmental disruptor in early mussel larvae at environmental concentrations. Moreover, these represent the first data on the possible mechanisms of action of TCS in developing larvae of a marine bivalve.

A review on source, bioaccumulation, degradability, and toxicity of TCS in aquatic environments reported an extremely wide range of concentrations of TCS in different water compartments, including about 100 ng/L in sea water, although higher concentrations (up to 362 ng/L) were found in coastal environments (Lydon et al. 2017). Based on ecotoxicity tests, TCS is considered very toxic for aquatic organisms, with EC_50_ < 1 mg/L and a PNEC in marine water of 0.169 µg/L (https://echa.europa.eu/registration-dossier/-/registered-dossier/12675/6/1). Although TCS has been banned from some products and replaced by other additives/biocides, according to the analysis of future scenarios for its global river export, TCS concentrations in coastal waters could double by 2050, exceeding PNECs in particular in certain geographic areas, due to fast population growth, increasing urbanization, and sewerage systems with poor wastewater treatment (van Wijnen et al. 2018).

Within this context of exposure, little information is available on the effects and mechanisms of action of TCS on development of marine invertebrates, whose early developmental stages are particularly sensitive to environmental perturbations (Przeslawski et al. 2015; Pandori and Sorte 2019). The results of the present work demonstrate that TCS affects early larval development in *M. galloprovincialis* in the 48-h standard larval assay, with an EC_50_ of 263.2 μg/L. This value is slightly higher but comparable with those previously reported in different bivalve species (Cortez et al. 2012; Di Poi et al. 2018; Tato et al. 2018; Rolton et al. 2022) (Table S2). However, our data, obtained in a generally wider concentration range than those of previous studies, also encompassing lower concentrations, indicate that TCS induced a significant decrease in normal D-larvae from 0.1 μg/L, followed by a plateau between 1 and 100 μg/L. In these conditions, TCS mainly induced shell malformations, followed by developmental arrest at higher concentrations. Although the results do not show significant changes in average shell size induced by TCS, as previously described for *M. galloprovincialis* (Rolton et al. 2022), distinct size distribution curves were observed in samples exposed to 10 and 100 μg/L with respect to controls, suggesting alterations in shell growth at concentrations lower than EC_50_ values.

Independent of ecotoxicity data, evidence is accumulating on the potential effects of TCS as an ED in humans and other organisms, acting through a variety of molecular mechanisms (reviewed in Alfhili and Lee 2019; Maksymowicz et al. 2021; Sinicropi et al. 2022). In fish, increasing evidence suggests that TCS can act as a developmental disruptor. TCS can induce reproductive toxicity, developmental deformities, and decreased hatching (reviewed in Dar et al. 2022). TCS has been shown to affect growth and differentiation in the zebrafish embryo (Stenzel et al. 2019; Liu et al. 2022; Phillips et al. 2022; Wang et al. 2022) and biochemical parameters and gene expression in the larvae of *Labeo rohita* (Sharma et al. 2021). In *Solea senegalensis*, TCS interfered with larval development and metamorphosis through downregulation of thyroid-related genes (Araújo et al. 2022).

The possible mechanisms of action of TCS in early mussel larvae were thus investigated by a transcriptomic approach, evaluating expression of selected groups of genes involved in different biological processes (shell biogenesis, neuroendocrine signaling, antioxidant and biotransformation response, ceramide metabolism, and apoptosis/proliferation). The results show that the effects of TCS on transcription of different genes were dependent on both the developmental stage and the exposure concentration. Although the observed changes were small, TCS exposure resulted in significant upregulation of a number of key genes in critical pathways of mussel early larval development from 24 hpf, a stage where the blueprint for shell calcification is established and tissue differentiation begins (i.e., development of the nervous system) (Miglioli et al. 2019, 2021a, 2021b).

TCS increased transcription of genes involved in CaCO_3_ deposition and mineralization, CA and EP (carbonic anhydrase and extrapallial protein), depending on the larval stage and concentration, and of Runx at 48 hpf. Runx is a gene belonging to a family of heteromeric transcription factors, involved in developmental cell differentiation, hemopoiesis, neurogenesis, immune response and biomineralization also in bivalves (Song et al. 2019; Zheng et al. 2019), and in response to acidification in *M. galloprovincialis* larvae (Kapsenberg et al. 2022). In contrast, TCS did not affect expression of tyrosinase, the key gene in organic matrix deposition during initial shell biogenesis (Miglioli et al. 2019), that has been shown to represent a sensitive target for EDs like BPA and TBBPA (Miglioli et al. 2021a, 2021b). The results indicate that genes involved in calcification, rather than in deposition of the organic matrix, can represent a target for the action of TCS in the growing shells of mussel larvae.

These data were supported by the observations of calcein staining, showing a general decrease in CaCO_3_ deposition in TCS-exposed larvae at 48 hpf. Interestingly, TCS also induced the “key-hole” phenotype (absence of calcification in the center of the valvae near the hinge) previously observed in mussel larvae grown under experimental ocean acidification (Kapsenberg et al. 2018) or exposed to TBBPA (Miglioli et al. 2021b). Moreover, PLM observations showed no birefringence in the shells of TCS-exposed larvae, indicating the absence of CaCO_3_ in the mineralized form (calcite or aragonite). A similar effect was previously observed in larvae exposed to nanoplastics (Balbi et al. 2017).

The effects of TCS on shell calcification and mineralization in mussel larvae are also in line with recent evidence indicating that TCS can affect osteogenesis in vertebrate systems. In mouse embryonic stem cell-derived osteoblasts, TCS affected skeletal differentiation through dysregulation of the BMP/ERK/Smad/Runx-2 signaling pathway (Cheng et al. 2019). In the zebrafish embryo, TCS (from 62.5 to 250 μg/L) induced developmental defect of craniofacial cartilage and impairment of skeletal mineralization, as well as changes in expression of marker genes of bone development; the effects were mediated by inhibition of the BMP signaling pathway (Wang et al. 2022). Our data support the hypothesis that the signaling pathways involved in CaCO_3_ deposition and mineralization can represent a significant target for TCS also in calcifying larvae of marine bivalves.

Shell morphology in TCS-exposed samples was further investigated by SEM. The results indicate that although many shells of larvae exposed to a wide range of concentrations of TCS (0.1–100 μg/L) had a straight hinge (one of the main morphological endpoints of the 48-h larval assay), they showed subtler malformations (in particular shell thinning and cracks at lower concentrations) that were not detectable by light microscopy. Since the dehydration protocol of sample preparation for SEM analysis was specifically developed in order to avoid any damage to normal D-veligers shells that are almost fully calcified (Balbi et al. 2016), the fractures observed in TCS-exposed samples indicate the presence of thinner, more fragile, or not fully calcified shells. Moreover, the presence of irregular patches formed by granules of 100–200 nm was observed, possibly representing ACC, as previously described during the transition from the trochophora to the D-veliger stage (Balbi et al. 2016). This indicates that TCS can affect CaCO_3_ mineralization from concentrations as low as 0.1 μg/L.

Overall, the results of detailed microscopical analysis of larval shells underline the limitations of the standard bivalve 48-h larval assay, where determination of normal larval phenotypes by standard light microscopy based on gross morphological parameters (D-shape, straight hinge), can underestimate the impact of contaminants on the key process of shell biogenesis at lower concentrations. Gene expression analyses obviously represent more sensitive indicators of toxicity, at the same time characterizing the molecular targets of contaminants, as shown in larvae of *M. californianus* for a legacy contaminant such as copper (Hall et al. 2020). This would particularly apply to those chemicals, including EDs, that may act as developmental disruptors in calcifying marine larvae at low concentrations through multiple and still unknown mechanisms of action.

In both adults and larvae, carbonate biominerals grow from a chemically complex aqueous environment in a biologically controlled space (Bots et al. 2012; Ma and Feng 2015; Ramesh et al. 2017). Within this membrane-closed microenvironment, the organism regulates the concentration and speciation of inorganic ions and organic components, all of which participate in particle formation, phase transitions, and growth of the final mineral polymorph (calcite and aragonite). TCS is a lipid soluble membranotropic agent that has been shown to induce membrane destabilization and to affect the activity of different membrane and mitochondrial ion transporters in a variety of eukaryotic cells (Alfhili and Lee 2019; Sinicropi et al. 2022). In this light, in mussel larvae, TCS may act within this microenvironment interfering with the complex processes of biomineralization, including ion transporters and shell related proteins (Ramesh et al. 2019). In CaCO_3_ biominerals of different marine invertebrates, including bivalves, the ACC phase is considered as a transient precursor of the crystallization pathway, which could be temporarily stabilized by organic macromolecules or trace elements, i.e., Mg^2+^ (Grünewald et al. 2022). In this light, the observed upregulation of biomineralization related genes, CA, EP, and Runx, may represent an attempt to counteract the effects of TCS on membrane functions during calcification.

With regards to data on other transcriptional responses, monoamine (serotonin and dopamine) pathways are involved in regulation of bivalve shell biogenesis by activating the expression of key genes essential for initial organic matrix deposition, i.e., tyrosinase (Liu et al. 2020; Canesi et al. 2022). We have previously shown that EDs like BPA and TBBPA interfere with these pathways in mussel early larvae (Miglioli et al. 2021a, 2021b). In contrast, TCS exposure did not significantly affect expression of monoamine receptors, in line with the absence of relevant effects on expression of genes involved in secretion of the shell organic matrix.

Among the multiple potential modes of action of TCS as an ED, interactions with different components of estrogen signaling and metabolism have been shown in mammals and fish (Alfhili et al. 2019; Cao et al. 2020; Maksymowicz et al. 2021; Kumar et al. 2021; Araújo et al. 2022). In mussel larvae, TCS induced a general increase in transcription of estrogen receptors MeER1 and MeER2, corresponding to vertebrate ERR and ER, respectively (Nagasawa et al. 2015), with MeER2 representing the most upregulated gene.

The ED effects of TCS have been also related to alteration of lipid metabolism in different animal models, from *Daphnia magna* to rodents (Sengupta et al. 2017; Huang et al. 2020). Lipids are a minor but significant components of the organic matrix of molluscan shell (representing from 0.8 to 2.9% and including polar lipids, sterols and triglycerides, fatty acids, and waxes) (Farre and Dauphin 2009; Marin et al. 2012). With regards to phospholipids, during *D. magna* growth, TCS induced alterations in metabolism of sphingomyelin and its metabolites, ceramides, sphingosine, or sphingosine-1-phosphate that play important roles in membrane cell signaling, growth, and development (Sengupta et al. 2017). We have recently observed that phospholipid profiles and ceramide related genes are modulated during *M. galloprovincialis* early development (Balbi et al. 2022). Ceramides are signaling molecules crucial in a variety of developmental processes, from proliferation to differentiation and apoptosis (Panevska et al. 2019) and in bone formation and remodeling (Qi et al. 2021). The results here obtained indicate that TCS affects the expression of ceramide related genes; in particular, aCDase and GC2, key genes in ceramide breakdown and glycosylation, respectively, were upregulated mostly in larvae at 24 hpf, suggesting a decrease in ceramide levels. Although the role of polar lipids in shell formation is still unexplored, the results support the hypothesis that ceramide metabolism may represent another significant target for the action of TCS in mussel development.

Finally, TCS exposure modulated the expression of genes involved in biotransformation at 48 hpf. Interestingly, GST represented the only downregulated gene, whereas the xenobiotic transporter ABCB was upregulated. This suggests that in mussel larvae, TCS, rather than undergoing phase II metabolization, could be partly eliminated by active extrusion processes. In contrast, no effects were observed on transcription of antioxidant enzymes SOD and CAT and of proliferation and apoptosis marker genes PCNA and p53. However, the absence of changes in gene expression does not rule out the induction of oxyradical production and oxidative damage or changes in specific activities of antioxidant enzymes induced by TCS, as previously observed in mussels (Canesi et al. 2007; Riva et al. 2012; Rolton et al. 2022).

Overall, the results provide a first insight on the mechanisms of action of TCS in early larval stages of *M. galloprovincialis*. Genes involved in shell calcification, estrogen signaling, and ceramide metabolism were those more clearly affected. Although at present it is not clear how these processes are intertwined, these data can contribute drawing a tentative adverse outcome pathway (AOP) for TCS in early mussel development.

Increasing knowledge on the impact and mechanisms of action of TCS in marine organisms is of environmental significance also when considering that TCS is one of the main emerging contaminant associated with ocean microplastic pollution (Beiras et al. 2021; Nobre et al. 2022). Moreover, several studies highlighted the negative impacts caused by TCS in different species of adult bivalves under a climate change scenario (Freitas et al. 2019; Costa et al. 2020; Maynou et al. 2021).

In developing larvae, information of the physiological mechanisms on shell biogenesis and calcification and how they can be affected not only by exposure to emerging contaminants, alone and in combination with changing environmental factors, will contribute to the ability to predict how these organisms can respond and adapt to environmental change.

### Supplementary Information

Below is the link to the electronic supplementary material.

Supplementary file1 (DOCX 459 KB)

## Data Availability

The data that support the findings of this study are available within the article.
