# Supplementary material for: The biocide triclosan as a potential developmental disruptor in Mytilus early larvae
Source: Environ Sci Pollut Res Int. 2023 Sep 20;30(48):106342–54. doi: 10.1007/s11356-023-29854-2 (PMC10579167; doi:10.1007/s11356-023-29854-2)
Supplement: Supplementary file 1 — Supplementary file1 (DOCX 459 KB) [file 11356_2023_29854_MOESM1_ESM.docx]

**SUPPLEMENTARY MATERIAL TO**

**The biocide Triclosan as a potential developmental disruptor in *Mytilus* early larvae**

Teresa Balbi^1,2^, Angelica Miglioli^3^, Michele Montagna^1^, Davide Piazza^1^, Beatrice Risso^1,3^, Remi Dumollard^3^ and Laura Canesi^1,2*^

^1^Department of Earth, Environmental and Life Sciences-DISTAV, University of Genoa, Italy

^2^National Biodiversity Future Center, Palermo, Italy

^3^Sorbonne Université/CNRS, Institut de la Mer, UMR7009 Laboratoire de Biologie du Développement, Villefranche-sur-Mer, France

*Corresponding Author

**Methods**

**Table S1. Primers sequences for qPCR.**

|  | **Gene** |  | **Primers** |
| --- | --- | --- | --- |
| **shell biogenesis** | **Chitin synthase** | ***CS*** | Fwd: AAC AGA AGC CAG GCA CTA TAT C  Rev: GTC AGA ACC AGC ACA GTA GTC |
|  | **Tyrosinase** | ***TYR*** | Fwd: CGA TTC TTT ATA CAT GAA ATC TGT G  Rev: AAA CCG TTA TAA CAA CGT GCT AA |
|  | **Carbonic anhydrase** | ***CA*** | Fwd: ACC AGA TGG TCT TGC AGT TT  Rev: TCA TCT CTG ACT GCT GCT AAT G |
|  | **Extrapallial protein** | ***EP*** | Fwd: TAA ACT CTG GAC ACG CAT ACC  Rev: GAG TCC CTC TTG GTG CAT ATT |
|  | **Runx transcription factor** | ***Runx*** | Fwd: CAC GTA TGC CAG ACC TTC CA  Rev: TCG CCA GAA TAA CGT TCG GT |
|  |  |  |  |
| **neuroendocrine** | **Serotonin receptor** | ***5-HTR*** | Fwd: CAG CTG CAA GAT CGA GGA TT  Rev: TGA AGC CAT CTT GAC TGA CG |
|  | **Dopamin receptor** | ***DR1*** | Fwd: GCC ATG ATA AGT GCG GTT TGG  Rev: CCA ATG GTT TGC CTC GAT CC |
|  | **Estrogen receptor 1** | ***MeER1*** | Fwd: TTA CGA GAA GGT GTG CGT TT  Rev: TCA CCA TAG GAA GGA TAT GT |
|  | **Estrogen receptor 2** | ***MeER2*** | Fwd: GGA ACA CAA AGA AAA GAA AGG AAG  Rev: ACA AAT GTG TTC TGG ATG GTG |
|  |  |  |  |
| **antioxidant**  **biotransformation** | **Superoxide dismutase** | ***SOD*** | Fwd: AGC CAA TGC AGA GGG AAA AGC AGA  Rev: CCA CAA GCC AGA CGA CCC CC |
|  | **Catalase** | ***Cat*** | Fwd: CGA CCA GAG ACA ACC CAC C  Rev: GCA GTA GTA TGC CTG TCC ATC C |
|  | **Glutathione-s-transferase** | ***GST*** | Fwd: TCC AGT TAG AGG CCG AGC TGA  Rev: CTG CAC CAG TTG GAA ACC GTC |
|  | **ABC transporter p-glycoprotein** | ***ABCB*** | Fwd: CAC CAT AGC CGA GAA CAT CC  Rev: CTC CAC GCT CTC CAA CTA G |
|  |  |  |  |
| **ceramide metabolism** | **Serine palmitoyltransferase-1** | ***SP2*** | Fwd: TTT GGT GCT GCT GGT GGT TA  Rev: ACT GCG GGA CAC ATT GTA GG |
|  | **1,3-ketodihydrosphingosine reductase** | ***KDSR2*** | Fwd: TCA AGT GGC ATA GGG AAG GC  Rev: CAA CCT TTG CAG CTT CGA GT |
|  | **Ceramide glucosyltransferase** | ***GC2*** | Fwd: TGT TGG AGT TGA CCC TCA CC  Rev: TGC TGA ATC CAT TTC ATC TTG GA |
|  | **Acid ceramidase** | ***aCDase*** | Fwd: CGA TTA TGG GAG GGG TTC GC  Rev: CCA TCT TCC TGA CTT GGC GT |
|  |  |  |  |
| **apoptosis**  **proliferation** | **Tumor suppression protein 53** | ***p53*** | Fwd: CAA ACT TGC TAA ATT TGT TGA AGA  Rev: TTG GTC CTC CTA CAC ATG AC |
|  | **Proliferating cell**  **nuclear antigen** | ***PCNA*** | Fwd: AGT TGG CCC AAA ATG CAA GT  Rev: TCT TGT GAC ATG GAC AGG CT |
|  |  |  |  |
| **housekeeping genes** | **Elongation factor-α1** | ***EF-α1*** | Fwd: CGT TTT GCT GTC CGA GAC ATG  Rev: CCA CGC CTC ACA TCA TTT CTT G |
|  | **Helicase** | ***HEL*** | Fwd: GCA CTC ATC AGA AGA AGG TGG C  Rev: GCT CTC ACT TGT GAA GGG TGA C |

**Results**

**Fig. S1 - Normalized size distribution of D-larvae in control samples and in samples exposed to TCS 10 and 100 μg/L.** A) length; B) width. Control: n=500; TCS 10 μg/L: n=350; TCS 100 μg/L: n=250.


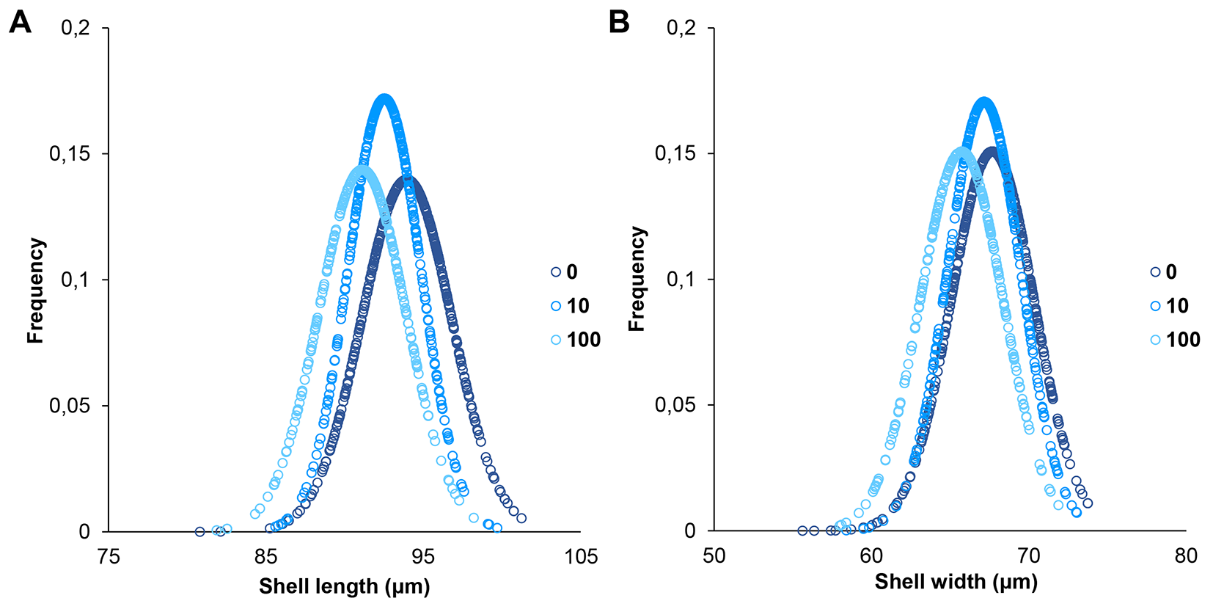


**Fig. S2 - Transcriptional profiles of selected gene products during early larval development in mussels.** Gene transcription was evaluated by qPCR in embryos of *M. galloprovincialis* grown under physiological conditions at 24 (trocophora) and 48 (D-veliger) hours post fertilization-hpf. Data, reported as log_2_-transformed relative expressions with respect to eggs, represent the mean ± SD (n=4). Expression of all transcripts was significantly changed at both 24 and 48 hpf with respect to eggs (1-way non-parametric ANOVA followed by the Mann-Whitney *U* test; p<0.05), except for CS and PCNA at 24 hpf *vs* eggs. Further statistical differences were evaluated between 24 and 48 hpf (* p<0.05, ** p<0.01, *** p<0.001, Mann-Whitney *U* test).


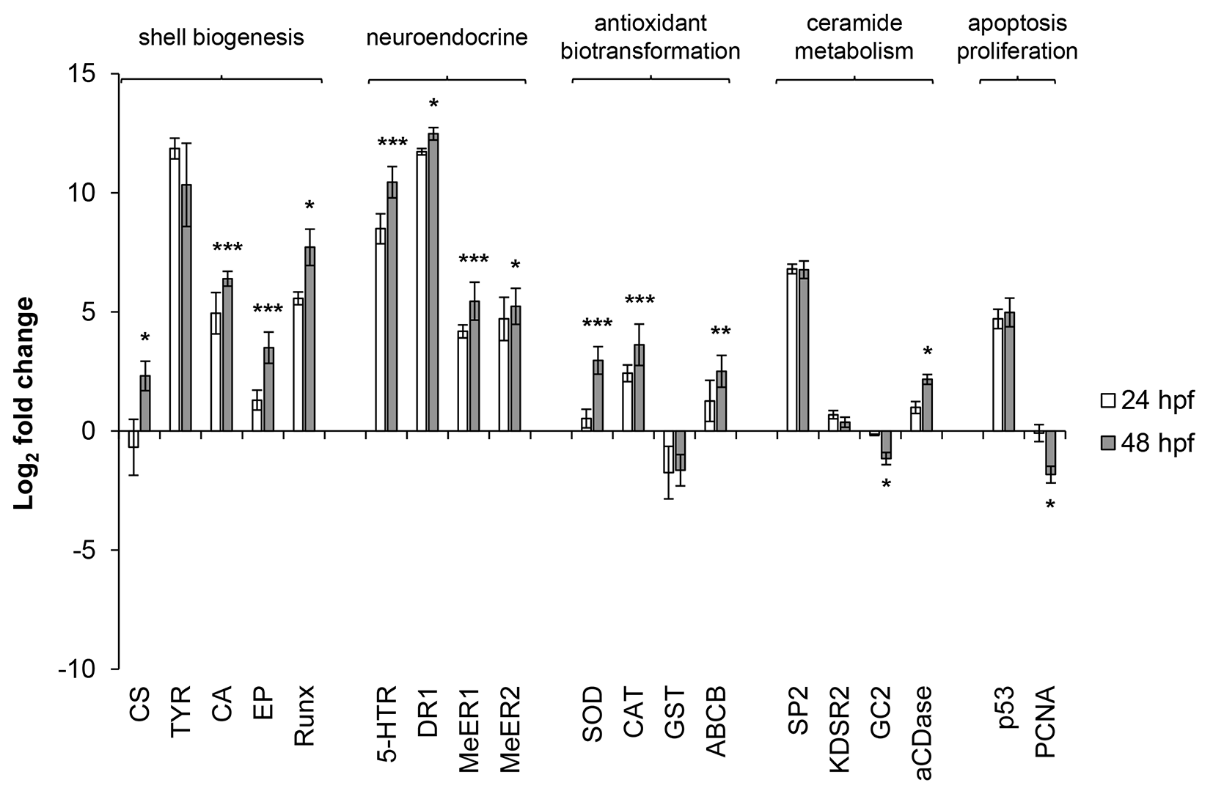


**Table S2 - Effects of TCS on early larval development of different species of marine bivalves evaluated in the embryotoxicity assay.** No Observed Effect Concentration (NOEC), Lowest Observed Effect Concentration (LOEC), and 50% effective concentrations (EC_50_) are reported (95% confidence intervals in brackets). Data are expressed as µg/L.

|  | **TCS concentration range (µg/L)** | **NOEC** | **LOEC** | **EC_50_** | **Reference** |
| --- | --- | --- | --- | --- | --- |
| *Perna viridis*  48 hpf | 10-320 | 56 | 76 | 135  (130-142) | Cortez et al., 2012 |
| *Mytilus galloprovincialis*  48 hpf | 50-250 | 120 | 160 | 149.8  (136.3-163.1) | Tato et al., 2018 |
| *Crassostrea gigas*  36 hpf | Not specified | 5 | 10 | 40 | Di Poi et al., 2018 |
| *Perna viridis*  *Mytilus galloprovincialis*  48 hpf | 0.5-500 | 114  53 | 194  106 | 213 (207-220)  94 (91.5-97) | Rolton et al., 2022 |
| *Mytilus galloprovincialis*  48 hpf | 0.001-1000 | 0.01 | 0.1 | 263.2  (139-498.2) | this work |
